# Caregiver-Reported Quality Measures and Their Correlates in Home Hospice Care

**DOI:** 10.1089/pmr.2020.0055

**Published:** 2020-07-07

**Authors:** Veerawat Phongtankuel, M.C. Reid, Sara J. Czaja, Jeanne Teresi, Joseph P. Eimicke, Jian X. Kong, Holly Prigerson, Ariel Shalev, Ritchell Dignam, Rosemary Baughn, Ronald D. Adelman

**Affiliations:** ^1^ Division of Geriatrics and Palliative Medicine, Weill Cornell Medicine, New York, New York, USA.; ^2^ Research Division, Hebrew Home at Riverdale, New York, New York, USA.; ^3^ Columbia University Stroud Center at New York State Psychiatric Institute, New York, New York, USA.; ^4^ Visiting Nurse Service of New York, New York, New York, USA.

**Keywords:** caregiving, end of life, hospice, palliative care

## Abstract

***Background:*** A majority of hospice care is delivered at home, with significant caregiver involvement. Identifying factors associated with caregiver-reported quality measures could help improve hospice care in the United States.

***Objectives:*** To identify correlates of caregiver-reported quality measures: burden, satisfaction, and quality of end-of-life (EoL) care in home hospice care.

***Design:*** A cross-sectional study was conducted from April 2017 through February 2018.

***Setting/Subjects:*** A nonprofit, urban hospice organization. We recruited caregivers whose patients were discharged from home hospice care. Eligible caregiver participants had to be 18 years or older, English-speaking, and listed as a primary caregiver at the time the patient was admitted to hospice.

***Measures:*** The (1) short version of the Burden Scale for Family Caregivers; (2) Family Satisfaction with Care; and (3) Caregiver Evaluation of the Quality of End-Of-Life Care.

***Results:*** Caregivers (*n* = 391) had a mean age of 59 years and most were female (*n* = 297, 76.0%), children of the patient (*n* = 233, 59.7%), and non-Hispanic White (*n* = 180, 46.0%). The mean age of home hospice patients was 83 years; a majority had a non-cancer diagnosis (*n* = 235, 60.1%), were female (*n* = 250, 63.9%), and were non-Hispanic White (*n* = 210, 53.7%). Higher symptom scores were significantly associated with greater caregiver burden and lower satisfaction with care; but not lower quality of EoL care. Caregivers who were less comfortable managing patient symptoms during the last week on hospice had higher caregiver burden, lower caregiver satisfaction, and lower ratings of quality of EoL care.

***Conclusion:*** Potentially modifiable symptom-related variables were correlated with caregiver-reported quality measures. Our study reinforces the important relationship between the perceived suffering/symptoms of patients and caregivers' hospice experiences.

## Introduction

Hospice care has become an integral part of care in the United States for many older adults and their families at the end of life (EoL), with more than 48% of all Medicare decedents receiving hospice services in 2017.^1^ When hospice is delivered at home, caregivers (the vast majority of whom are family members) play a critically important role in the patient's care. They help manage patients' symptoms and comorbid conditions, provide emotional support, and assist with day-to-day caregiving duties.^2^ As a result, caregivers are an integral member of the patient's care team and a key source of information for assessing the quality of hospice care.

Although there are various ways to measure quality of EoL care, no consensus indicators have been agreed on.^3^ Caregiver-reported quality measures such as perceived burden, satisfaction, and quality of EoL care are aspects of care that are important and commonly discussed in the literature, but they have not been thoroughly explored in the context of home hospice care.^4,5^ Further, research examining caregiver-reported quality measures and their correlates in the home hospice setting is limited. In one study conducted with 44 caregivers receiving hospice care, researchers found that caregivers who were married or were taking care of a patient with end-stage renal disease reported lower family satisfaction with care scores.^6^

Another, more recent study analyzed responses from the Consumer Assessment of Healthcare Providers and Systems (CAHPS) survey, a nationwide hospice quality measure initiated by the Centers for Medicare and Medicaid Services (CMS) and reported by caregivers. The investigators found that type of payer for hospice, caregiver education, and language spoken at home were most predictive of CAHPS survey scores.^7^

To our knowledge, no study has examined correlates of caregiver-reported quality measures such as caregiver burden (short version of the Burden Scale for Family Caregivers [BSFC-s]), caregiver satisfaction (Family Satisfaction with Care [FAMCARE-2]), and quality of EoL care (Caregiver Evaluation of the Quality of End-Of-Life Care [CEQUEL]) in this setting. Understanding correlates of these three measures could provide insight into factors that place caregivers at risk for a poor/difficult hospice experience and suggest approaches to improve quality of care.

The primary objective of our study was to identify correlates of three caregiver-reported quality measures: caregiver burden, quality of EoL care, and caregiver satisfaction in a large urban home hospice program. Given that comfort is a principal goal of hospice care, we hypothesized that higher patient symptom burden (i.e., higher Edmonton Symptom Assessment Scale [ESAS] scores), as reported by the caregiver, would be independently associated with higher caregiver burden, lower satisfaction with care, and lower quality of EoL care after adjusting for relevant covariates.

## Methods

### Study design and setting

This cross-sectional study collected data on caregiver-reported quality measures in the home hospice setting. The Institutional Review Boards (IRBs) of Weill Cornell Medicine and the Visiting Nurse Service of New York approved the study.

The Visiting Nurse Service of New York Hospice and Palliative Care (VNSNY-HPC) organization is a nonprofit hospice that serves more than 1000 patients daily and delivers hospice care to patients in the New York City area. In addition to providing home visits by an interdisciplinary team of physicians, nurses, social workers, and spiritual care counselors, VNSNY-HPC provides enrolled patients a medication kit for pain and symptom management and educational materials that describe available support services. A hospice on-call team provides round-the-clock phone service whereby a hospice nurse, nurse practitioner, or physician may be dispatched to the home based on the needs of the patient and family.

### Sample assembly

During the study period (April 2017 through February 2018), VNSNY-HPC staff generated a weekly list that contained the names of all patients discharged from home hospice in the preceding week. Additional information provided by the hospice agency included patient demographic data (age, gender, race/ethnicity, hospice diagnosis—cancer vs. non-cancer, length of stay, reason for discharge), a home hospice utilization variable (use of continuous home hospice care during the last week on hospice), as well as caregiver contact information (name, address, phone number).

Caregivers received a mailed letter introducing the study and informing them to expect a call in two weeks from a member of the research team. Eligible caregiver participants had to be 18 years or older, English speaking, and listed as a primary caregiver (e.g., family or friend) at the time the patient was admitted to the VNSNY-HPC service. A trained research assistant called potential participants, described the study, and obtained verbal consent from interested and eligible individuals. Of the 1848 caregivers contacted, 804 (44%) did not answer the phone after three attempts to contact them, 653 (35%) declined participation, and 391 (21%) completed the phone survey interview.

### Data collection

A semistructured interview guide was used to guide the phone interview after consent was obtained. Caregivers received a $25 gift card for their participation.

### Dependent variables: Quality measures

Three quality measures were administered to caregivers during the phone interview. Caregiver burden was measured by using the BSFC-s.^8^ The internal consistency (ordinal alpha) estimate for this sample was 0.891. Caregivers' appraisals of their satisfaction with care and the quality of EoL care were assessed by using the FAMCARE-2^9^ scale and CEQUEL scale,^10^ respectively. The FAMCARE-2 is a 17-item scale that measures the degree to which family members are satisfied with the health care received by both the patient and the family with respect to information giving, availability of care, psychological care, and physical patient care. This measure has been widely used in palliative care research, specifically in the palliative care setting.^9,11^ The CEQUEL scale is a 13-item instrument that includes unique markers on perceived suffering and prolongation of death. Lower CEQUEL scores are associated with poor bereavement outcomes.^12^ The internal consistency (ordinal alpha) estimate for this sample was 0.826. Because of missing data in the outcome variables, the analytic sample sizes were less than the total sample, and they varied across outcomes.

### Patient covariates

Patient-level data included age, gender, race/ethnicity, hospice diagnosis (cancer vs. non-cancer), reason for discharge, use of continuous home hospice care during the last week on hospice, and length of hospice stay. Caregivers served as proxy respondents to measure patient symptom prevalence and level of severity. Symptoms were assessed by using the ESAS. Caregivers were asked to recall whether the patient experienced any of the nine symptoms included in the ESAS (i.e., pain, shortness of breath, nausea, tiredness, drowsiness, lack of appetite, depression, anxiety, well-being) and to rate their intensity on a 0-to-10 scale during the patient's last week on hospice. The ESAS has evidence of good psychometric properties,^13^ and it been used in numerous studies of patients with terminal illnesses and those at the EoL.^14–16^ Although obtaining assessments from patients would be the gold standard, given that most patients were entering the last stage of dying, this was not feasible. However, there is established evidence for the validity of using proxy respondents to assess patient symptoms at the EoL.^17–19^

### Caregiver covariates

The following caregiver demographic data were collected during the telephone interviews: age, gender, race/ethnicity, relationship with the patient, education level, and average hours of caregiving provided per day during the last seven days on home hospice. We measured the caregiver's comfort in managing patients' symptoms by asking participants, “How would you rate your level of comfort managing (patient's name) symptoms during the last week on home hospice care?” Choices ranged from 1 (very comfortable) to 5 (very uncomfortable).

All data obtained from the medical record and through survey questions were entered into Research Electronic Data Capture (REDCap), a secure web application for building and managing databases.

### Statistical approach

Bivariate analyses were conducted to examine patient and caregiver characteristics associated with each individual quality measure. Point-biserial correlation coefficients were used for binary variables and Pearson correlation coefficients were employed for ordinal and continuous variables.

Variables included in multivariable regression analysis were based on clinical importance and also statistical significance in bivariate analysis. Analyses were conducted to evaluate the unique association between the BSFC-s, CEQUEL, FAMCARE-2, and covariates. The pre-specified alpha level was set at 0.05 for each of the outcome variables. Independent regression models were performed, because the correlations of the three outcomes were relatively low (0.07–0.37). Collinearity diagnostics were examined, and sensitivity analyses were conducted removing potentially collinear variables.

Bivariate analyses were performed by using IBM SPSS Statistics version 25 (IBM Corp, 2016), and multivariable analyses were performed by using SAS (SAS Institute, Inc., *SAS Version 9.4*. Cary, NC: SAS Institute, Inc.; 2015).

## Results

Demographic data for patients and caregivers are presented in Tables 1 and 2. The mean age of patients was 83 years; a majority had a non-cancer diagnosis (*n* = 235, 60.1%), were female (*n* = 250, 63.9%), and non-Hispanic White (*n* = 210, 53.7%). The average length of stay in hospice was 98 days with a median of 33 days. Caregivers had a mean age of 59 years and most were female (*n* = 297, 76.0%), children of the patient (*n* = 233, 59.7%), and had a college education or greater (*n* = 271, 76.1%). Caregivers reported providing an average of 14 hours of patient care per day during the patient's last week on hospice. Death was the major reason for discharge (*n* = 351, 89.8%), followed by hospitalization (*n* = 24, 6.1%), and finally others (*n* = 16, 4.1%).

**Table 1.** Patient Characteristics (*n* = 391)

	n (%)	Mean (SD)
Patient age	391	83 (14)
Patient gender		
Male	141 (36)	—
Female	250 (64)	—
Patient race/ethnicity		
White	210 (54)	—
Black	63 (16)	—
Hispanic	75 (19)	—
Asian	29 (7)	—
Other/undisclosed	14 (4)	—
Hospice diagnosis		
Cancer	156 (40)	—
Non-cancer	235 (60)	—
Length of stay (days)	391	98 (178)
Reason for discharge from hospice		
Death	351 (90)	—
Hospitalization	24 (6)	—
Other	16 (4)	—
Received continuous home care during the last week on hospice	35 (9)	—

SD, standard deviation.

**Table 2.** Caregiver Characteristics (*n* = 391)

	n (%)	Mean (SD)
Caregiver age	351	59.3 (12.5)
Caregiver gender		
Male	94 (24)	—
Female	297 (76)	—
Caregiver race/ethnicity		
White	180 (46)	—
Black	57 (15)	—
Hispanic	75 (19)	—
Asian	28 (7)	—
Other	9 (2)	—
Not specified	42 (11)	—
Caregiver relationship with patient		
Child	233 (60)	—
Spouse	69 (18)	—
Relative	59 (15)	—
Grandchild	11 (3)	—
Friend	11 (3)	—
Parent	3 (1)	—
Caregiver education level		
High school	84 (24)	—
College	168 (47)	—
Graduate school	103 (29)	—
Average hours per day spent caregiving during the last week on hospice	363	14.4 (9.5)
Comfort managing symptoms^a^	349	2.3 (1.4)
Caregiver rated Edmonton Symptom Assessment Scale^b^	362	51.2 (17.4)

^a^ Scale from 1 to 5 with 1 = very comfortable and 5 = very uncomfortable.

^b^ Nine-item scale with higher score indicates greater distress. Range 0–90.

### Caregiver burden (BSFC-s) scores and correlates

The mean BSFC-s score (Table 3) was 15.5 (standard deviation [SD] = 5.5). Table 4 shows bivariate correlation coefficients and *p*-values for BSFC-s score and patient, caregiver, and hospice utilization variables. Higher ratings of caregiver burden scores were associated with patient hospitalization (*r* = 0.150, *p* ≤ 0.01), younger caregiver age (*r* = −0.189, *p* ≤ 0.001), higher ESAS scores (*r* = 0.288, *p* ≤ 0.001), and caregivers who were less comfortable managing patient symptoms (*r* = 0.176, *p* ≤ 0.001).

**Table 3.** Caregiver-Rated Quality Measures

	n	Mean (SD)
BSFC-s^a^	366	15.5 (5.5)
FAMCARE-2^b^	355	29.6 (13.2)
CEQUEL scale^c^	338	22.4 (3.0)

^a^ Higher score indicates more burden; possible range 0–30.

^b^ Higher score indicates worse satisfaction; possible range 17–85.

^c^ Higher score indicates better perceived quality of care; possible range 13–26.

BSFC-s, short version of the Burden Scale for Family Caregivers; CEQUEL, Caregiver Evaluation of the Quality of End-Of-Life Care; FAMCARE-2, Family Satisfaction with Care.

**Table 4.** Bivariate Analysis of Quality Measures in Home Hospice Population

	BSFC-s (n = 359)	FAMCARE-2 (n = 350)	CEQUEL (n = 332)
	r	r	r
Patient age	−0.075	−0.077	−0.013
Patient gender			
Male			
Female	−0.016	−0.079	0.085
Patient race/ethnicity^a^			
White	0.022	−0.014	−0.027
Black	−0.008	0.015	0.017
Hispanic	0.035	−0.030	0.019
Asian	−0.079	0.048	−0.003
Other/undisclosed^b^			
Hospice diagnosis			
Cancer			
Non-cancer	0.012	−0.101	−0.039
Length of stay (days)	0.002	−0.073	−0.107^*^
Reason for discharge from hospice^a^			
Death	−0.196^***^	−0.168^***^	0.095
Hospitalization	0.150^**^	0.192^***^	−0.046
Other^b^			
Received continuous home care during the last week on hospice	−0.071	−0.037	0.011
Caregiver age	−0.189^***^	−0.085	−0.031
Caregiver gender			
Male			
Female	0.101	−0.015	0.057
Caregiver race/ethnicity^a^			
White	−0.003	−0.007	−0.015
Black	−0.038	−0.060	0.044
Hispanic	0.056	−0.030	0.040
Asian	−0.074	0.037	0.001
Other^b^			
Not specified	0.073	0.061	−0.110^*^
Caregiver relationship with patient^a^			
Child	0.100	−0.031	0.139^**^
Spouse	−0.020	0.060	−0.089
Relative	−0.118^*^	−0.051	−0.083
Grandchild^b^			
Friend^b^			
Parent^b^			
Caregiver education level^a^			
High school	−0.050	−0.143^**^	0.053
College	0.028	0.027	−0.009
Graduate school	0.025	0.102	−0.045
Average hours per day spent caregiving during the last week on hospice	0.022	0.023	−0.017
ESAS	0.288^***^	0.200^***^	−0.110^*^
Comfort managing symptoms^c^	0.176^***^	0.359^***^	−0.192^***^

BSFC-s: Higher scores are associated with greater caregiver burden. FAMCARE-2: Higher scores are associated with lower satisfaction. CEQUEL: Lower scores are associated with poor bereavement.

^a^ Dummy variables used as reference group.

^b^ Not computed due to sparse data.

^c^ Scale from 1 to 5 with 1 = very comfortable and 5 = very uncomfortable.

^*^ *p* ≤ 0.05, ^**^p ≤ 0.01, ^***^p ≤ 0.001.

ESAS, Edmonton Symptom Assessment Scale.

Table 5 shows the results of the linear regression model predicting BSFC-s score. Higher caregiver burden scores were associated with higher ESAS scores (estimate = 0.074, *p* < 0.001), patients who did not die on hospice (estimate = −3.288, *p* < 0.001), older caregivers (estimate = 0.080, *p* = 0.008), and caregivers who were less comfortable managing patient symptoms (estimate = 0.413, *p* = 0.050).

**Table 5.** Linear Regression Analysis for Quality Measures in Home Hospice Population

	BSFC-s (n = 359)			FAMCARE-2 (n = 350)			CEQUEL scale (n = 332)		
	Estimate	SE	p	Estimate	SE	p	Estimate	SE	p
Intercept	15.127	3.039	<0.001	18.980	7.157	0.008	23.567	1.893	<0.001
ESAS (higher score indicates greater distress)	0.074	0.017	<0.001	0.084	0.039	0.034	−0.017	0.010	0.103
Patient's age	0.014	0.030	0.643	0.084	0.070	0.231	−0.013	0.018	0.459
Patient female	0.472	0.608	0.438	−1.331	1.416	0.348	0.506	0.362	0.163
Patient white	0.421	0.599	0.482	−2.091	1.398	0.136	−0.076	0.355	0.831
Cancer hospice diagnosis	−1.084	0.657	0.100	1.765	1.536	0.252	0.275	0.395	0.488
Length of stay	0.001	0.002	0.877	−0.004	0.004	0.312	−0.002	0.001	0.022
Death discharge reason	−3.288	0.951	0.001	−5.191	2.199	0.019	0.844	0.697	0.227
Received continuous home care during last week on hospice	1.161	0.956	0.225	0.754	2.257	0.739	0.389	0.556	0.484
Caregiver age	0.080	0.030	0.008	−0.104	0.071	0.140	0.010	0.018	0.559
Caregiver female	1.063	0.674	0.116	−0.234	1.569	0.881	0.346	0.396	0.383
Caregiver spouse	0.844	0.990	0.395	3.027	2.319	0.193	−0.825	0.588	0.162
Caregiver education	0.367	0.391	0.349	2.187	0.912	0.017	−0.064	0.229	0.782
Number of hours with patient per day	0.017	0.033	0.618	−0.004	0.077	0.957	−0.008	0.020	0.696
Comfort managing symptoms during last week on hospice care^a^	0.413	0.210	0.050	2.987	0.490	<0.001	−0.322	0.127	0.012

BSFC-s: Higher scores are associated with greater caregiver burden. FAMCARE-2: Higher scores are associated with lower satisfaction. CEQUEL: Lower scores are associated with poor bereavement.

^a^ Scale from 1 to 5 with 1 = very comfortable and 5 = very uncomfortable.

SE, standard error.

### Caregiver satisfaction (FAMCARE-2) scores and correlates

The mean FAMCARE-2 score (Table 3) was 29.6 (SD = 13.2). Table 4 shows bivariate correlation coefficients and *p*-values for FAMCARE-2 score and patient, caregiver, and hospice utilization variables. Lower satisfaction scores were associated with patients who were hospitalized (*r* = 0.192, *p* ≤ 0.001), higher ESAS score (*r* = 0.200, *p* ≤ 0.001), caregivers who had greater than a high school education (*r* = −0.143, *p* ≤ 0.01), and caregivers who were less comfortable managing patient symptoms (*r* = 0.359, *p* ≤ 0.001).

Table 5 shows the results of the linear regression model predicting FAMCARE-2 score. Lower caregiver satisfaction scores were associated with higher ESAS scores (estimate = 0.084, *p* = 0.034), patients who did not die on hospice (estimate = −5.191, *p* = 0.019), caregivers with higher education (estimate = 2.187, *p* = 0.017), and caregivers who were less comfortable managing patient symptoms (estimate = 2.987, *p* < 0.001).

### Quality of EoL care (CEQUEL) scores and correlates

The mean CEQUEL score (Table 3) was 22.4 (SD = 3.0). Table 4 shows bivariate correlation coefficients and *p*-values for CEQUEL score and patient, caregiver, and hospice utilization variables. Lower quality of EoL care scores were associated with longer length of stay (*r* = −0.107, *p* ≤ 0.05), caregivers who did not specify their race (*r* = −0.110, *p* ≤ 0.05), caregivers who were children of the patient (*r* = 0.139, *p* ≤ 0.05), higher ESAS score (*r* = −0.110, *p* ≤ 0.05), and caregivers who were less comfortable managing patient symptoms (*r* = −0.192, *p* ≤ 0.01).

Table 5 shows the results of the linear regression model predicting CEQUEL score. Lower ratings of quality of EoL care were associated with longer hospice length of stays (estimate = −0.002, *p* = 0.022) and caregivers who were less comfortable managing patient symptoms (estimate = −0.322, *p* = 0.012). Contrary to our primary study hypothesis, CEQUEL scores were not significantly related to symptom burden at the multivariate level.

## Discussion

Our study examined the correlates of caregiver-reported quality measures in home hospice care. We found that caregiver comfort in managing patient symptoms during the last week on hospice was associated with all three quality measures examined: caregiver burden, caregiver satisfaction, and quality of EoL care. We also found that both higher caregiver-reported symptom scores and caring for patients who did not die in hospice were associated with higher caregiver burden and lower satisfaction with care.

We used the BSFC-s to measure caregiver burden in this study. The mean score for our sample was 15.5 (SD = 5.5). To provide some context, a study of caregivers of dementia patients in Germany found a lower mean BSFC-s score of 10.2 (SD = 8.0).^8^ Overall satisfaction was high in our sample, which is consistent with many studies examining satisfaction with hospice care.^20^ Lastly, our reported quality of EoL care scores (mean = 22.4, SD = 3.0) are similar to a previous study conducted by Higgins and Prigerson looking at CEQUEL scores in advanced cancer patients and their caregivers (mean = 23.6, SD = 2.2).^10^

We hypothesized that higher caregiver-reported patient symptom scores (i.e., ESAS) would be associated with all three outcomes based on our clinical experience caring for this population and past work linking symptoms to poor patient outcomes such as hospitalization.^21,22^ We did find that higher caregiver-reported ESAS scores were associated with two of the quality measures, caregiver burden and satisfaction with care, but not with quality of EoL care. This may be a result of the questions on the CEQUEL survey, which captures aspects of quality other than patient symptom burden such as prolongation of death and shared decision making.

Along the same lines, we did find that caregivers of patients who died on hospice compared with those discharged alive (i.e., hospitalized or discharged for other reasons) had lower caregiver burden scores and higher caregiver satisfaction ratings. Reducing symptoms and avoidable care transitions at the EoL are difficult challenges to address and further research aimed at treating symptoms, supporting caregivers, and finding solutions to reduce unnecessary hospice transitions is needed.

In our regression analysis, caregivers' comfort level managing symptoms during the last week on hospice was independently associated with all three quality measures. This finding is of interest and seems particularly pertinent to home hospice care. Caregivers spend a significant amount of time caring for patients.^2^ We speculate that caregivers who are more comfortable managing symptoms feel they are providing better palliative care, which may lead to better reported quality measures. Further research is needed to validate and measure caregiver efficacy in providing EoL care and understand its longitudinal impact on outcomes.

Based on our analysis, interventions to help improve caregivers' knowledge and skills in understanding and managing symptoms may be appropriate.^23,24^ We have shown in our qualitative work^25^ that caregivers expressed the need for more knowledge around what to expect at the EoL. Past interventions conducted by Cagle et al.^23^ and Campbell and McErlane^24^ to address pain and dyspnea, respectively, can be building blocks to support home hospice caregivers. It will be important for future work to better describe the spectrum of caregiver roles in home hospice care, as well as the social and educational supports they receive, which may vary considerably depending on the underlying diagnosis of the care recipient, family makeup, and hospice organization, to provide more tailored approaches to help support them.

From a clinical standpoint, our study reinforces the important relationship between perceived suffering/symptoms of patients and caregivers' home hospice experiences. Although we know that hospice improves patients' quality of life^26^ and many caregivers report high satisfaction with care, studies also have found that burdensome symptoms are still prevalent.^27,28^ Hospices should continue to strive to improve how symptoms are both evaluated and managed. In terms of future research, we believe that advancement in detecting and treating signs and symptoms is one area that warrants further study. Further, given the critically important role that caregivers play in this setting, finding ways to support and educate caregivers, whether it is through better access to clinical supervision, support through advancing technologic aides (e.g., telemedicine, online educational videos), or other models of care delivery, are important to understand, develop, and rigorously test.

Our study has several limitations. First, we interviewed caregivers instead of patients to measure ESAS scores. Although it would have been preferable to obtain ESAS data from patients, we had concerns about recruitment (e.g., being able to obtain patient consent and adequate sample size), along with concerns about the potential burden of administering surveys to patients at the EoL. We, therefore, elected to collect proxy data from caregivers postdischarge.^17–19^ In addition, caregivers' comfort level managing patient symptoms and recall bias may have impacted reporting of symptoms. Second, despite our multiple attempts to reach potential participants, our refusal and nonresponse rates were high, which highlights the recruitment challenges researchers face when conducting EoL/hospice research,^29^ and may have biased the sample. We did find variations in the average length of stay (98 vs. 82 days), proportion of patients who were discharged due to death (90% vs. 86%), and proportion of patients with a cancer diagnosis (40% vs. 48%) between respondents and non-respondents. Third, although we had a diverse sample in terms of race/ethnicity, a majority of participants were highly educated with either college or graduate school education. Lastly, we only recruited from one nonprofit, urban hospice organization, which may not reflect the national makeup of caregivers and patients receiving home hospice care.

In conclusion, our study showed that caregiver-reported quality measures (i.e., caregiver burden, caregiver satisfaction, and quality of EoL care) were associated with symptom-related variables. Further research and strategies are needed to improve symptom management for patients and support caregivers in this area to improve quality of care in the home hospice setting.

## Abbreviations Used

BSFC-s: short version of the Burden Scale for Family Caregivers
CAHPS: Consumer Assessment of Healthcare Providers and Systems
CEQUEL: Caregiver Evaluation of the Quality of End-Of-Life Care
CMS: Centers for Medicare and Medicaid Services
EoL: end of life
ESAS: Edmonton Symptom Assessment Scale.
FAMCARE-2: Family Satisfaction with Care
IRBs: Institutional Review Boards
REDCap: Research Electronic Data Capture
SD: standard deviation
SE: standard error
VNSNY-HPC: Visiting Nurse Service of New York Hospice and Palliative Care
